# Supplementary material for: Performance of glomerular filtration rate equations using serum creatinine in children with congenital anomalies of the kidney and urinary tract
Source: PLoS One. 2026 Jan 6;21(1):e0340649. doi: 10.1371/journal.pone.0340649 (PMC12774354; doi:10.1371/journal.pone.0340649)
Supplement: Attachment — Submitted filename: Response to Reviewers.docx [file pone.0340649.s002.docx]

PONE-D-25-32018

Performance of Glomerular Filtration Rates Equations Using Creatinine Serum in Children with Congenital Anomalies of Kidney and Urinary Tract

PLOS ONE

Dear Editor in Chief PlosOne,

We thank you and the reviewers for the insightful and constructive feedback on our manuscript titled:

**“Performance of Glomerular Filtration Rate Equations Using Serum Creatinine in Children with Congenital Anomalies of the Kidney and Urinary Tract.”**

We have carefully revised the manuscript in response to all suggestions. The major revisions are summarized below:

1. **Expanded justification of equation selection**
   We added a detailed explanation of why bedside Schwartz and EKFC were chosen, and clarified the limited availability of cystatin C and CKiD U25 in Indonesian clinical practice.
2. **Clarified clinical procedures and addressed potential bias**
   We specified that serum creatinine was obtained on the same day as Tc-99m DTPA and discussed possible selection bias related to mGFR procedures.
3. **Re-examined and explained discordant cases**
   We assessed highly discordant mGFR–eGFR pairs, explained likely clinical reasons, and added a sensitivity analysis, noting that conclusions remained unchanged.
4. **Improved characterization of CAKUT cohort**
   We added detailed CAKUT subtype distribution and further stratified mGFR categories (G1 vs G2).
5. **Clarified the age distribution and avoided inaccurate implications of “age of onset.”**
6. **Added definition of P30 and corrected table references**
   P30 is now clearly defined, and incorrect table citations have been fixed.
7. **Improved figure resolution and table layout**
   All figures have been replaced with high-resolution versions and tables 2–3 have been reformatted for better readability.
8. **Edited for clarity and English grammar**
   Several sentences were reworded for accuracy, and the reviewer-suggested title has been adopted.

We believe these revisions substantially strengthen the manuscript by improving clarity, methodological transparency, and clinical relevance.

We sincerely thank the reviewers for their time and expertise. We hope the revised manuscript will now be suitable for publication in PLOS ONE.

Kind regards,

Aryati

**Journal Requirements:**

When submitting your revision, we need you to address these additional requirements.

1. Please ensure that your manuscript meets PLOS ONE's style requirements, including those for file naming. The PLOS ONE style templates can be found at

<https://journals.plos.org/plosone/s/file?id=wjVg/PLOSOne_formatting_sample_main_body.pdf> and [https://journals.plos.org/plosone/s/file?id=ba62/PLOSOne_formatting_sample_title_authors_affiliations.pdf](https://track.editorialmanager.com/CL0/https:%2F%2Fjournals.plos.org%2Fplosone%2Fs%2Ffile%3Fid=ba62%2FPLOSOne_formatting_sample_title_authors_affiliations.pdf/1/010f0199dd87c3e0-d440a69d-5349-481d-8fd8-94729bf7b7bf-000000/zhM6Pjd7YV0s0C-vpPG01JjR21FqabEjW_DE6VV1ydo=232)

Response:

Thank you for the information regarding the style template requirements. We have adjusted it according to the PLOS ONE template

2. We note that the grant information you provided in the ‘Funding Information’ and ‘Financial Disclosure’ sections do not match.

When you resubmit, please ensure that you provide the correct grant numbers for the awards you received for your study in the ‘Funding Information’ section.

Response:

Thank you for the correction. We have revised the funding information

3. Thank you for stating in your Funding Statement:

[AA was supported by Universitas Airlangga, Surabaya, East Java, Indonesia, grant number 345/UN3.LPPM/PT.01.03/2024 under Penelitian Unggulan Airlangga (PUA) 2024 initiative and FRM received support by Soetomo Hospital, Surabaya, East Java, Indonesia, with grant number 100.3.3/25.1/102.6/2024 by Penelitian Unggulan Hospital Scheme.].

Please provide an amended statement that declares *all* the funding or sources of support (whether external or internal to your organization) received during this study, as detailed online in our guide for authors at <http://journals.plos.org/plosone/s/submit-now>.  Please also include the statement “There was no additional external funding received for this study.” in your updated Funding Statement.

Please include your amended Funding Statement within your cover letter. We will change the online submission form on your behalf.

Response:

Thank you for the information. We have amended the statement regarding the research funding sources and included the statement: ‘There was no additional external funding received for this study.’ We have also communicated this update in the cover letter

4. Thank you for stating the following in your manuscript:

[This work was supported by Universitas Airlangga, Surabaya, East Java, Indonesia, grant number 345/UN3.LPPM/PT.01.03/2024 under Penelitian Unggulan Airlangga (PUA) 2024 initiative and our work received support by Soetomo Hospital, Surabaya, East Java, Indonesia, with grant number 100.3.3/25.1/102.6/2024 by Penelitian Unggulan Hospital Scheme.]

We note that you have provided funding information that is currently declared in your Funding Statement. However, funding information should not appear in the Acknowledgments section or other areas of your manuscript. We will only publish funding information present in the Funding Statement section of the online submission form.

Please remove any funding-related text from the manuscript and let us know how you would like to update your Funding Statement. Currently, your Funding Statement reads as follows:

[AA was supported by Universitas Airlangga, Surabaya, East Java, Indonesia, grant number 345/UN3.LPPM/PT.01.03/2024 under Penelitian Unggulan Airlangga (PUA) 2024 initiative and FRM received support by Soetomo Hospital, Surabaya, East Java, Indonesia, with grant number 100.3.3/25.1/102.6/2024 by Penelitian Unggulan Hospital Scheme.]

Please include your amended statements within your cover letter; we will change the online submission form on your behalf.

Response:

We have removed the funding-related text from the manuscript and included the following statement: ‘AA was supported by Universitas Airlangga, Surabaya, East Java, Indonesia, grant number 345/UN3.LPPM/PT.01.03/2024 under the Penelitian Unggulan Airlangga (PUA) 2024 initiative, and FRM received support from Soetomo Hospital, Surabaya, East Java, Indonesia, with grant number 100.3.3/25.1/102.6/2024 under the Penelitian Unggulan Hospital Scheme.

5. In the online submission form, you indicated that [Data are available from the Dr Soetomo Academic Hospital Data Access (contact via [kontak.rsudrsoetomo@jatimprov.go.id](mailto:kontak.rsudrsoetomo@jatimprov.go.id)) for researchers who meet the criteria for access to confidential data.].

All PLOS journals now require all data underlying the findings described in their manuscript to be freely available to other researchers, either 1. In a public repository, 2. Within the manuscript itself, or 3. Uploaded as supplementary information.

This policy applies to all data except where public deposition would breach compliance with the protocol approved by your research ethics board. If your data cannot be made publicly available for ethical or legal reasons (e.g., public availability would compromise patient privacy), please explain your reasons on resubmission and your exemption request will be escalated for approval.

Response:

We have presented the data with the hospital’s permission, using anonymized information, and it can be reviewed in the supplementary information

6. Please include a separate caption for each figure in your manuscript.

Response:

Thank you. We have separated the captions for each figure in the manuscript

7. If the reviewer comments include a recommendation to cite specific previously published works, please review and evaluate these publications to determine whether they are relevant and should be cited. There is no requirement to cite these works unless the editor has indicated otherwise. 

Response:

Thank you. This study does not compare with the CKID U25, which has not yet been adopted in Indonesia; however, we have added information regarding the CKID U25 analysis in the results and discussion sections


[Note: HTML markup is below. Please do not edit.]

Reviewers' comments:

Reviewer's Responses to Questions

**Comments to the Author**

1. Is the manuscript technically sound, and do the data support the conclusions?

The manuscript must describe a technically sound piece of scientific research with data that supports the conclusions. Experiments must have been conducted rigorously, with appropriate controls, replication, and sample sizes. The conclusions must be drawn appropriately based on the data presented.

Reviewer #1: Partly

Reviewer #2: Yes

Response:

We have addressed all of the major and minor concerns raised by Reviewer 1.

2. Has the statistical analysis been performed appropriately and rigorously?

Reviewer #1: I Don't Know

Reviewer #2: Yes

Response:

We have addressed all major and minor concerns from Reviewer 1, including the addition of an explanation for the P30 definition.

3. Have the authors made all data underlying the findings in their manuscript fully available?

The [PLOS Data policy](http://track.editorialmanager.com/CL0/http:%2F%2Fwww.plosone.org%2Fstatic%2Fpolicies.action%23sharing/1/010f0199dd87c3e0-d440a69d-5349-481d-8fd8-94729bf7b7bf-000000/MbgnbijxaJRveNYbvAsWCW0W52zuVTYql83R_0t_pmY=232) requires authors to make all data underlying the findings described in their manuscript fully available without restriction, with rare exception (please refer to the Data Availability Statement in the manuscript PDF file). The data should be provided as part of the manuscript or its supporting information, or deposited to a public repository. For example, in addition to summary statistics, the data points behind means, medians and variance measures should be available. If there are restrictions on publicly sharing data—e.g. participant privacy or use of data from a third party—those must be specified.

Reviewer #1: Yes

Reviewer #2: Yes

Response:

Thank you, and we appreciate both reviewers

4. Is the manuscript presented in an intelligible fashion and written in standard English?

PLOS ONE does not copyedit accepted manuscripts, so the language in submitted articles must be clear, correct, and unambiguous. Any typographical or grammatical errors should be corrected at revision, so please note any specific errors here.

Reviewer #1: Yes

Reviewer #2: Yes

Response:

Thank you, and we appreciate both reviewers

5. Review Comments to the Author

Please use the space provided to explain your answers to the questions above. You may also include additional comments for the author, including concerns about dual publication, research ethics, or publication ethics. (Please upload your review as an attachment if it exceeds 20,000 characters)

**Reviewer #1:** In this study by Marpaung et al, two different eGFR calculators, the bedside Schwartz and EKFC are compared to measured GFR using 99mTc DTPA in a cohort of children with CAKUT from their institution. They find that both eGFR equations correlated with mGFR but that the bedside Schwartz tended to overestimate GFR while the EKFC underestimated GFR. This could provide helpful information for clinicians in guiding interpretation of creatinine-based eGFR equations for children with CAKUT. However, there are some major concerns that should be addressed, including whether serum creatinine was measured at the same time as the 99mTc DTPA study.

Major Concerns:
1. I am curious why the authors chose the bedside Schwartz and EKFC equations as there are many other calculating equations that have been developed. In fact, many pediatric nephrologists have shifted to using the recently developed CKiD U25 creatinine and cystatin C based eGFR formulas (Pierce Kidney Int 99:948, 2021). It would be informative to compare the CKiD U25 creatinine based equation with the bedside Schwartz and EKFC equation. If the authors have cystatin C data, addition of a combined creatinine-cystatin C based calculation would also be informative.

Response:

We appreciate this important point regarding the choice of GFR estimating equations.

In Indonesia, the **bedside Schwartz equation** remains the standard equation recommended in National and Ministry of Health pediatric CKD guidance and is the routine formula used in our hospital for clinical decision-making in children. Thus, any validation in our local CAKUT population must, as a first priority, address how well this widely used equation reflects measured GFR (mGFR) in our patients. The **EKFC equation** was selected because it represents a contemporary “full age spectrum” creatinine-based formula, designed to harmonize GFR estimation from childhood into adulthood and increasingly recommended in European settings. It uses age (and not height) as a primary covariate, which is attractive for laboratory-based reporting and for regions where height measurement is not systematically captured in electronic records. Evaluating whether EKFC can be safely adopted in our CAKUT population has immediate practical implications for laboratories in Indonesia.

Although we agree that the **CKiD U25 equations** (creatinine-only and creatinine–cystatin C) show excellent performance in CKD cohorts, they have **not yet been incorporated into Indonesian practice guidelines** or routine laboratory reporting.

More importantly, **serum cystatin C is not routinely available** in our institution and in most Indonesian hospitals. It is substantially more expensive than creatinine and currently not covered by the national health insurance, which severely limits feasibility for routine clinical use in CAKUT patients. For the purpose of this retrospective study, we therefore did not have cystatin C measurements to compute combined creatinine–cystatin C equations.

Finally, we fully agree with the reviewer that **validation of CKiD U25 creatinine-based in Indonesian children**, including defining appropriate population-specific creatinine coefficients (analogous to population-specific “Q” values in EKFC), is an important and logical next step. We have add the **CKiD U25 creatinine evaluation performance I this manuscript.**

(page 3 line 1-2, page 4 line 21, page 5 line 3-4)

2. Given that this is a retrospective study, I am wondering if it is routine for all children with CAKUT to undergo mGFR measurements? Could there be selection bias in favor of discordant mGFR vs. eGFR in your cohort due to the inclusion of only patients who underwent 99mTc DTPA?

Response:

Thank you for raising this important issue of potential selection bias.

In our center, **Tc-99m DTPA mGFR is recommended for all children with CAKUT** when accurate GFR measurement is required for clinical decisions (e.g., pre-intervention assessment, significant structural abnormalities, or concern for CKD progression). During the study period, all newly diagnosed CAKUT patients were **offered** Tc-99m DTPA mGFR measurement as part of routine work-up.

Approximately **19% of children with CAKUT did not undergo Tc-99m DTPA**, primarily due to: parental refusal of nuclear medicine procedures, logistic constraints, or scheduling issues rather than suspicion of discrepant eGFR. We agree that **some degree of selection bias cannot be excluded.** Those who accepted a nuclear medicine procedure may differ in unmeasured ways (e.g., disease severity, family preference, or socioeconomic factors) from those who declined.

To address this, we have now:

1. **Explicitly acknowledged possible selection bias** in the **Limitations** section and clarified that our findings strictly apply to CAKUT children for whom mGFR was clinically indicated and successfully obtained.
2. Emphasized that the **clinical indication for Tc-99m DTPA was driven by the presence of CAKUT and need for accurate GFR**, rather than by prior suspicion of a discrepancy between eGFR and mGFR. Therefore, while selection bias may exist, we believe it is unlikely to be systematically biased toward highly discordant eGFR/mGFR pairs.

We have added clarifying sentences on this point in the **Methods**  and **Limitations** sections.

(page 4 line 5-10, page 12 line 11-23)

3. Was the serum creatinine used to calculate the bedside Schwartz and EKFC eGFR taken at the same time that the 99mTc DTPA was done?

Response:

Yes. We appreciate the chance to clarify this important methodological detail.

In our institutional protocol for nuclear renography**, serum creatinine is drawn immediately before the Tc-99m DTPA injection,** during the same visit and on the same day as the mGFR measurement.

For this study, **only creatinine values obtained as part of the same visit as Tc-99m DTPA** were used to calculate bedside Schwartz and EKFC eGFR. No creatinine measurements from earlier or later visits were included.

We have now explicitly stated this in the **Methods – measured GFR** section to make the temporal alignment between creatinine and Tc-99m DTPA measurements clear.

(page 4 line 10-12)

4. While some discrepancy in eGFR calculators is expected, it is surprising to me the number of very discordant cases (Table 3). If I am understanding the table correctly, it appears that there are two individuals with a G1 mGFR and G4 or G5 EKFC. Similarly there are a few individuals with G1 or G2 EKFC and G4 or G5 mGFR. There are also a few individuals with a G1 bedside Schwartz and G4 and G5 mGFR. Can you please comment on these discrepancies as this is very surprising. Were these individuals with complex medical conditions and very low muscle mass, for example, and, if so, should they be excluded in your analysis?

Response:

We agree that these **highly discordant category pairs** merit careful scrutiny and explanation.

1. **Re-examination of discordant cases**
   - We revisited the raw data for all children whose **KDIGO GFR categories differed by three or more stages** between mGFR and eGFR (for both equations). These represent a **very small minority** of the cohort.
   - The clinical profiles of these outliers shared features that are **known to challenge creatinine-based GFR estimation,** including:
     - **Very low or very high muscle mass** relative to body size (e.g., children with severe growth delay or chronic illness),
     - **Extremely abnormal serum creatinine values** out of proportion to stable structural renal damage, suggesting either non–steady-state kidney function or possible intercurrent illness around the time of measurement, and
     - Early infancy with rapidly changing GFR and creatinine generation, where the maturation of renal function and creatinine production is highly dynamic.
2. **Sensitivity analysis excluding extreme discordant cases**
   - We performed a **sensitivity analysis excluding these extreme outliers** and recalculated bias, RMSE, concordance, and P30 for both equations.
   - As expected, removal of these few outliers **slightly improved some performance indices, but did not materially change the overall conclusions:**
     - EKFC remained more accurate than bedside Schwartz, particularly in children >2 years.
     - The direction of bias (underestimation by bedside Schwartz, overestimation by EKFC) was preserved.
3. **Rationale for retaining these cases in the primary analysis**
   - From a clinical perspective, such extreme discordances are exactly the situations in which clinicians most need to understand the limitations of creatinine-based equations.
   - Excluding them would make the equations appear more accurate than they are in real-world practice, especially in children with unusual body composition or non–steady-state kidney function, which are not rare in CAKUT and pediatric nephrology.

We have now:

- Added a **paragraph in the Discussion** specifically describing these highly discordant cases and their likely explanation (body composition, non–steady state, extremes of age), and
- Mentioned that a **sensitivity analysis excluding these outliers** does not change the overall ranking of equation performance (EKFC vs bedside Schwartz), while we deliberately present the full cohort analysis as our primary result to reflect real-world use.

(page 10 line 10-21, page 11 line 1-14)

5. CAKUT is a very heterogeneous group of disorders. It would be useful to provide some baseline demographic information regarding the type of CAKUT (even if just broken into things like solitary kidney, dysplasia/hypodysplasia, VUR, PUV, etc)

Response:

We agree that CAKUT is heterogeneous and that the **distribution of underlying anomalies** is informative.

- We have now **expanded Table 1** to include an additional row summarizing **CAKUT subtypes**, grouped into clinically relevant categories such as:
  - Obstructive including: ureteropelvic junction obstruction (UPJ), VUJ,PUV etc
  - Non obstructive including renal agenesis, renal cyst and etc

Because our study was not powered to compare equation performance across each morphological subtype, we present these data **descriptively** and explicitly state that **subgroup analyses by CAKUT subtype** will require larger multi-center cohorts. This clarification has been added to the **Results (Baseline characteristics)** and **Discussion** sections.

(Table 1 page 20 and page 9 line 19-20)

6. The figure legends are difficult to read. Please load higher quality figures.

Response:

Thank you for pointing this out.

- We have **re-exported all figures at higher resolution**, ensuring that axis labels, confidence intervals, and legends are easily readable.
- Each figure now has a **separate, clear caption** describing the regression plots and Bland–Altman analyses, as requested by the journal.

(Figures 1-6)

Minor Concerns:
1. Methods, page 6 and Conclusion, page 9: In general, the accepted definition of CKD is either the presence of “kidney damage for > 3 months as defined by structural or functional abnormalities with or without decreased GFR” or “GFR < 60 mL/min/1.73m2 for > 3 months, with or without kidney damage). Thus by definition, children with CAKUT have chronic kidney disease; those with eGFR > 90 will have CKD 1. Thus in paragraph 3 on page 9, in the sentence “The prevalence of CKD in this CAKUT cohort was CKD 8.7%,” should specify which stage of CKD you mean.

**Response:**
We appreciate this clarification. We fully agree with the KDIGO definition of CKD as either **kidney damage for >3 months or GFR <60 mL/min/1.73 m² for >3 months.**

- In this retrospective study, we only had **single-time-point mGFR data** for each child; we therefore cannot confirm the 3-month chronicity requirement for all individuals.
- To avoid implying that we measured CKD incidence per se, we have revised the text to state that:
  - **8.7% of the cohort had an mGFR <60 mL/min/1.73 m² at the time of evaluation**, corresponding to at least CKD stage 3 by GFR criteria, rather than claiming a prevalence of CKD as a diagnosis.

This clarification has been incorporated into the **Results** and **Discussion**

**(page 10, line 15-17)**

2. Results, page 7, first full paragraph: Do you mean Table 3 instead of Table 2?

**Response**:

Thank you for your concern**.** Yes, this was an error. We have corrected the table reference to the appropriate table in the revised manuscript.

3. Can you explain the meaning of “P30” in the body of the document.

**Response:**
We agree that P30 needed a clear definition.

- We now define P30 at its **first mention** in the Methods and again in the Results as:

“P30, defined as the percentage of eGFR values within ±30% of the corresponding mGFR value.”

This definition has been added to the **Statistical Analysis** section and referenced in the **Results** when reporting P30 values.

(page 6 line 5-7)

4. Conclusion, page 9: I don’t think this study was necessarily designed to study the incidence or age of onset of CAKUT in your population, especially given that 19% of patients with CAKUT were excluded due to incomplete data. Also, I am surprised that the age of onset was 7 years but perhaps this is due to lower prevalence or quality of prenatal imaging studies in Indonesia?

**Response:**
We agree and appreciate this important nuance.

- Our study design indeed **does not allow us to estimate the incidence** or true age of onset of CAKUT. We only know the **age at which children were evaluated and underwent mGFR measurement** at our center.
- We have revised the relevant sentences to:
  - Avoid referring to “age of onset”, and instead describe **“age at evaluation in our tertiary referral hospital”.**
  - Emphasize that the median age of 7 years likely reflects **referral patterns and timing of recognition** (including limited access to or utilization of prenatal and early postnatal imaging in parts of Indonesia), rather than the biological onset of CAKUT.

These clarifications have been added to the **Discussion.**

(page 10 line 7-8)

5. While the majority of the manuscript is well-written, there are certain sentences that should be reviewed and edited (e.g. Results, page 6, paragraph 3; Discussion, page 7, paragraph 1 – suggest changing “early diagnosis of kidney disorders” to “early and accurate diagnosis of impaired GFR”, also “Scwartz” is spelled incorrectly here and “recently equation” does not make grammatical sense; Conclusion, page 9, paragraph 2, “may affected to this varied result”). Also in the title, suggest “Performance of Glomerular Filtration Rate Equations Using Serum Creatinine in Children with Congenital Anomalies of the Kidney and Urinary Tract.”

**Response:**
Thank you for these helpful editorial suggestions.

- We have carefully **reviewed and edited the manuscript for English language clarity**, including the sentences highlighted:
  - “early and accurate diagnosis of impaired GFR” instead of “early diagnosis of kidney disorders,”
  - correction of “Scwartz” to “Schwartz,”
  - revision of grammatically awkward phrases such as “recently equation” and “may affected to this varied result.”
- We have **adopted the reviewer’s suggested title,** which more precisely describes the study:

“Performance of Glomerular Filtration Rate Equations Using Serum Creatinine in Children with Congenital Anomalies of the Kidney and Urinary Tract.”

**Reviewer #2:** This study composed of 276 children with CAKUT compared the bedside Schwartz and EKFC creatinine-based equations against measured GFR using technetium-99m diethylenetriaminepentaacetic acid (Tc-99mDTPA) clearance. The results showed that in children over 2 years old, EKFC demonstrated more accuracy compared to the bedside Schwartz. Below is my suggestions that may improve the quality of this study:
1. The proportion of patient with mGFR Tc-99mDTPA ≥ 60 ml/mnt/1.73 is 91.3%. Could the authors further classified these patients such as ≥ 90 ml/mnt/1.73, 60~89 ml/mnt/1.73?

**Response:**
We agree that a more granular description of the higher GFR range is useful.

- We have now **further stratified mGFR according to KDIGO categories:**
  - G1: ≥90 mL/min/1.73 m²,
  - G2: 60–89 mL/min/1.73 m²,
  - and G3a–G5 as previously defined.
- These distributions are now presented explicitly in:
  - the **Baseline Characteristics (Table 1)** for mGFR, and
  - the **classification comparison table (Table 3)** for mGFR vs EKFC and mGFR vs bedside Schwartz.

This allows readers to see how many CAKUT patients in our cohort fall into G1 versus G2 at the time of measurement.

(table 1, page 20)

2. The layout of Table 2 and Table 3 needs to be improved.

**Response:**
We appreciate this suggestion.

- We have **reformatted Tables 2 and 3** to improve readability:
  - In Table 2, we now separate clearly the results for:
    - all ages vs >2 years and
    - bedside Schwartz vs EKFC,
      including median eGFR, bias, slope, intercept, RMSE, correlation, and P30.
  - In Table 3, the contingency tables for **KDIGO categories** (mGFR vs EKFC and mGFR vs bedside Schwartz) have been aligned and labeled more clearly, with:
    - row and column totals,
    - categorical agreement percentages,
    - and weighted kappa values prominently displayed.

We believe these changes substantially improve interpretability.

6. PLOS authors have the option to publish the peer review history of their article ([what does this mean?](https://track.editorialmanager.com/CL0/https:%2F%2Fjournals.plos.org%2Fplosone%2Fs%2Feditorial-and-peer-review-process%23loc-peer-review-history/1/010f0199dd87c3e0-d440a69d-5349-481d-8fd8-94729bf7b7bf-000000/lY3nvCfTP248Ym6raH7PEgVfq21PZSzXpLwXBjQjx6k=232)). If published, this will include your full peer review and any attached files.

If you choose “no”, your identity will remain anonymous but your review may still be made public.

**Do you want your identity to be public for this peer review?** For information about this choice, including consent withdrawal, please see our [Privacy Policy](https://track.editorialmanager.com/CL0/https:%2F%2Fwww.plos.org%2Fprivacy-policy/1/010f0199dd87c3e0-d440a69d-5349-481d-8fd8-94729bf7b7bf-000000/14yLNLSobUHAHai1Mc05NAdEKPsdjEocU-6IuHvqAzU=232).

Reviewer #1: No

Reviewer #2: No

[NOTE: If reviewer comments were submitted as an attachment file, they will be attached to this email and accessible via the submission site. Please log into your account, locate the manuscript record, and check for the action link "View Attachments". If this link does not appear, there are no attachment files.]

While revising your submission, please upload your figure files to the Preflight Analysis and Conversion Engine (PACE) digital diagnostic tool, [https://pacev2.apexcovantage.com/](https://track.editorialmanager.com/CL0/https:%2F%2Fpacev2.apexcovantage.com%2F/1/010f0199dd87c3e0-d440a69d-5349-481d-8fd8-94729bf7b7bf-000000/GKoRfMDUSmXvJ2Y7z7HzdXnvX-BvX0XmITHzY-RaCpw=232). PACE helps ensure that figures meet PLOS requirements. To use PACE, you must first register as a user. Registration is free. Then, login and navigate to the UPLOAD tab, where you will find detailed instructions on how to use the tool. If you encounter any issues or have any questions when using PACE, please email PLOS at [figures@plos.org](mailto:figures@plos.org). Please note that Supporting Information files do not need this step.
