# Supplementary material for: Performance of glomerular filtration rate equations using serum creatinine in children with congenital anomalies of the kidney and urinary tract
Source: PLoS One. 2026 Jan 6;21(1):e0340649. doi: 10.1371/journal.pone.0340649 (PMC12774354; doi:10.1371/journal.pone.0340649)
Supplement: Attachment — Submitted filename: Response to Reviewer1 .docx [file pone.0340649.s003.docx]

**PONE-D-25-32018R1**

Performance of Glomerular Filtration Rates Equations Using Creatinine Serum in Children with Congenital Anomalies of Kidney and Urinary Tract

PLOS ONE

Dear Academic Editor and Reviewer,

We sincerely thank Reviewer 1 for the positive assessment of our revision and for the helpful suggestion to clarify the inclusion criteria in the Methods section. In response, we have expanded the “Population Study” subsection to explicitly describe (i) how CAKUT was defined and operationalized in our electronic medical record (EMR) search, (ii) the eligible age range, (iii) the exact study time window, and (iv) the relationship between the excluded cases and absence of paired 99mTc-DTPA mGFR measurements. We have also added a brief statement in the Methods reinforcing that the excluded 19% primarily reflects children without a completed 99mTc-DTPA assessment (and therefore without mGFR), complementing our discussion of this issue in the Limitations section.

**Reviewer #1, Comment:**

“My only suggestion is to be more clear in the methods section regarding the inclusion criteria... including how they defined ‘CAKUT’, what age ranges they considered for inclusion, and the exact time range that was considered. In addition, the authors say that 65 (19%) were excluded due to incomplete data – were these the 19% that did not undergo 99mTc DTPA? If so, I think it would be helpful to mention this in the methods section...”

**Response:**

We agree and appreciate this suggestion. We have revised the Methods to make the inclusion criteria explicit and reproducible. Specifically, we now state that we identified all pediatric patients with a *primary* diagnosis of CAKUT documented in the EMR within the study period and then included only those with a complete paired dataset for same-day serum creatinine and 99mTc-DTPA measured GFR (mGFR), alongside key demographic variables required for eGFR calculation.

• Definition of CAKUT: We now explicitly define CAKUT in the Methods as congenital structural anomalies of the kidneys and/or urinary tract documented in the EMR, and we clarify that CAKUT subtypes were abstracted from the clinical diagnosis and categorized as obstructive vs non-obstructive phenotypes (as summarized in Table 1).

• Age range: We now explicitly restate that eligible participants were children aged 6 months to 18 years at the time of 99mTc-DTPA renography.

• Time window: We now specify the exact time window used for EMR identification and data extraction (from January 1, 2017 through March 15, 2024; date of data access/extraction).

• Excluded 19% and 99mTc-DTPA: Yes—these 65 excluded cases were primarily those without a completed 99mTc-DTPA study and therefore lacking mGFR, and/or missing other required variables (e.g., height or same-day creatinine). We now state this explicitly in the Methods and briefly summarize common reasons (e.g., parental refusal of nuclear medicine procedures, logistic constraints, or scheduling issues), consistent with the Limitations section.

**Text added/updated in the manuscript (Methods – “Population Study” subsection):**

“This retrospective cohort study used electronic medical record (EMR) data from Dr. Soetomo Academic General Hospital to identify pediatric patients with a *primary* diagnosis of congenital anomalies of the kidney and urinary tract (CAKUT) from January 1, 2017 to March 15, 2024 (date of data extraction). CAKUT was operationalized as a congenital structural anomaly of the kidneys and/or urinary tract recorded as the primary clinical diagnosis in the EMR; subtypes were abstracted from the treating clinicians’ documented diagnosis and grouped as obstructive and non-obstructive CAKUT phenotypes (Table 1). Eligible participants were children aged 6 months to 18 years who underwent technetium-99m diethylenetriaminepentaacetic acid (99mTc-DTPA) renography for measured GFR (mGFR) and had serum creatinine assessed on the same day immediately preceding renography. We excluded records with missing data needed to compute eGFR or evaluate outcomes (age, sex, height, serum creatinine, mGFR, and urine albumin-to-creatinine ratio [ACR]). Of 341 CAKUT records screened, 65 (19%) were excluded because a complete paired dataset was not available, most commonly because 99mTc-DTPA was not performed, and/or because other key variables (e.g., height or same-day creatinine) were missing.”

We hope these revisions address the reviewer’s request for greater methodological clarity and improve reproducibility of the study.

Sincerely,

Aryati Aryati

(Corresponding Author)
